# Primary Endocrine Therapy in Older Women with Breast Cancer

**DOI:** 10.1007/s13670-017-0223-z

**Published:** 2017-10-19

**Authors:** R. M. C. Pepping, J. E. A. Portielje, W. van de Water, N. A. de Glas

**Affiliations:** 1 Department of Internal Medicine, Haga Hospital, The Hague, The Netherlands; 2 Department of Clinical Oncology, Leiden University Medical Centre, P.O. Box 9600, 2300 RC Leiden, The Netherlands; 3 Department of Surgery, Leiden University Medical Centre, Leiden, The Netherlands

**Keywords:** Primary endocrine therapy, Breast cancer, Elderly, Surgery, Geriatric oncology, Geriatric assessment

## Abstract

**Purpose of Review:**

Breast cancer incidence increases with age. In recent years, primary endocrine therapy has been increasingly used as a treatment option for frail elderly women with breast cancer, although surgery is still the guideline-recommended treatment. In this review, we discuss the evidence for primary endocrine therapy versus surgical treatment in older women with early breast cancer.

**Recent Findings:**

Both randomised controlled trials and recent observational studies showed a favourable progression-free survival but not overall survival for surgery plus adjuvant endocrine therapy versus primary endocrine therapy. Information about quality of life with either treatment strategy is so far lacking. Deciding who is fit for surgery and has sufficiently long life expectation to be at risk of disease progression can be supported by performing an individual geriatric assessment.

**Summary:**

This review suggests that primary endocrine therapy is a reasonable alternative to primary surgery in frail older women with breast cancer. Future studies should focus on the long-term effects on quality of life and physical functioning.

## Introduction

In recent years, the number of older adults with cancer is increasing due to ageing of the population in developed countries. Around 35% of women with breast cancer is 65 years or older at diagnosis [1]. Older women with breast cancer comprise a heterogeneous group, as many women have concomitant diseases and geriatric syndromes [2]. As a consequence, the risk of dying from other causes than breast cancer (the so-called competing mortality) increases strongly with age [3•]. This complicates treatment decisions, since some women with a long life expectancy may benefit from aggressive anti-cancer treatment, while others may benefit more from a more conservative strategy. Since the early 1990s, there has been an increasing interest in treating clinically localised breast cancer with tamoxifen only in elderly women [4]. The distribution of cancer subtype differs between elderly and younger patients, with an increasing proportion of oestrogen receptor (ER)-positive tumours with increasing age [5, 6].

The most recent guideline of the International Society for Geriatric Oncology (SIOG) and European Society of Breast Cancer Specialists (EUSOMA) advises to perform either breast-conserving surgery with radiotherapy or a mastectomy in all older women with non-metastasised breast cancer [7••].

However, previous randomised clinical trials in breast cancer included relatively few older women [7••, 8, 9]. In addition, it has been shown that older women who were included in randomised trials were not representative of the general older population, as they had less comorbidity, more favourable tumour characteristics and better outcomes [10••]. In addition, geriatric parameters and the risk of competing mortality are often not addressed in these trials, although these are strong predictors for long-term outcome [11]. As a consequence, the evidence base for treatment of older women with breast cancer is limited, and deviance from current guidelines may be justified in many cases.

In this report, the evidence for treatment with primary endocrine therapy (PET) in older women with breast cancer will be reviewed and discussed.

## Randomised Clinical Trials

There have been several randomised clinical trials over the last few decades that studied primary endocrine therapy compared to surgical treatment, with or without adjuvant endocrine therapy. The most important ones are already discussed in the review of Hind et al. [12••]. These studies are summarised below, including (when available) long-term follow-up results. All recent studies will be discussed separately for PET versus surgery alone and for PET versus surgery plus endocrine therapy (Tables 1 and 2).

**Table 1:** Studies on PET compared to surgery alone

Ref., author	Number of patients	Therapy	Follow-up	Outcome	ER status evaluated
[13] Fentiman et al.	164	Radical mastectomy or tamoxifen 20 mg daily	Median of 10 years	OS HR (95% CI) 1.11 (0.75–1.65) PFS HR (95% CI) 0.55 (0.39–0.77)	No
[14] Kenny et al.	131	Wedge mastectomy or tamoxifen 40 mg daily	Median of 5 years	OS HR (95% CI) 1.06 (0.59–1.92) PFS not calculated	No
[15, 16] Gazet et al.	200	Mastectomy or tamoxifen 20 mg daily	6 years	OS HR (95% CI) 0.75 (0.44–1.26) PFS not calculated	No
[5] Chakrabati et al.	131	Wedge mastectomy or tamoxifen 20 mg daily	20 years	OS *P* = 0.4457 PFS *P* = <0.001	No

*ER* oestrogen receptor, *OS* overall survival, *HR* hazard ratio, *CI* confidence interval, *PFS* progression-free survival

**Table 2:** Studies on PET compared to surgery followed by endocrine therapy

Ref., author	Number of patients	Therapy	Follow-up	Outcome	ER status evaluated
[18, 19] Mustacchi et al.	474	Mastectomy plus tamoxifen 20 mg daily or tamoxifen 20 mg daily alone Both groups received a 160-mg loading dose on day 1	13 years	OS HR (95% CI) 0.98 (0.77–1.25) PFS HR (95% CI) 0.65 (0.53–0.81)	Yes
[20] Fennessy et al.	455	Mastectomy plus tamoxifen 40 mg or tamoxifen 40 mg daily	Median of 12.7 years	OS HR (95% CI) 0.78 (0.63–0.96) PFS not calculated	Unknown
[21] Johnston et al.	153	Mastectomy plus tamoxifen 20 mg daily or tamoxifen 20 mg daily	Median of 6.5 years	OS at 5 years: *P* = 0.206 OS at 10 years: *P* = 0.802 PFS not calculated	Yes
[17] Wilsher et al.	147	Mastectomy plus tamoxifen 20 mg daily or tamoxifen 20 mg daily	Median of 3 years	OS HR (95% CI) 0.80 (0.73–2.32) PFS not calculated	Yes

*ER* oestrogen receptor, *OS* overall survival, *HR* hazard ratio, *CI* confidence interval, *PFS* progression-free survival

### Primary Endocrine Therapy Compared to Surgical Treatment Alone

Fentiman et al. [13] included 164 women of 70 years and older, who were randomised between a radical mastectomy and tamoxifen 20 mg daily. They showed that the risk of local progression was significantly increased in the tamoxifen group, with an astonishing 62 versus 11% in the surgery group, with a median follow-up of 10 years. Although this study was underpowered, they found no significant difference regarding overall survival. Similarly, Kenny et al. [14] found an improved local control in the wedge mastectomy group with 38% of relapse in the surgery group compared to 81% in the tamoxifen group with a daily dose of 40 mg. However, no differences in overall survival were observed.

Gazet et al. [15] included 200 women ≥ 70 years of age with oestrogen (ER)-positive tumours and concluded that at 6 years of follow-up, surgery alone was more effective in terms of overall survival and local control than tamoxifen alone. Hence, primary endocrine treatment with tamoxifen alone was considered a delay to the definitive treatment with surgery and not adequate therapy. However, the final report of Gazet et al. [16] with a follow-up of 28 years showed that survival between the groups of surgery or tamoxifen did not differ at all. Also, after crossover on recurrence from tamoxifen to surgical treatment, there was no difference in survival. Furthermore, two thirds of their patients died eventually of another cause than breast cancer.

Wilsher’s et al. [17] compared 20 mg tamoxifen twice daily with mastectomy in a randomised controlled trial of 131 women. Forty-two percent of women had a complete response at 6 months in the tamoxifen alone group and no significant difference in survival was observed between both groups. However, an inferior progression control was noted with tamoxifen alone, 81 versus 38% with surgery, in line with the study from Kenny et al.

Finally, Chakrabati et al. [5] published the final results of a randomised clinical trial (*n* = 131) of mastectomy versus tamoxifen alone, after 20 years of follow-up, with only two patients still alive (both with tamoxifen alone). They found no difference in time to regional progression. But, more importantly, they also found no statistically significant difference between both groups in terms of overall survival.

In summary, the meta-analysis of Hind et al. combined the results of above-mentioned studies and concluded with regard to overall survival that surgery alone is not superior to tamoxifen treatment in the elderly with breast cancer (hazard ratio (HR) 0.98, 95% confidence interval (CI) 0.74–1.30, *P* = 0.9). However, the meta-analysis showed a significant difference in progression-free survival favouring surgery over tamoxifen alone (HR 0.55, 95% CI 0.39–0.77, *P* = 0.0006). Long-term follow-up of these studies supported these findings.

### Primary Endocrine Therapy Compared to Surgical Treatment Followed by Adjuvant Endocrine Therapy

Mustacchi et al. [18, 19] compared primary endocrine therapy with tamoxifen alone to surgery with adjuvant tamoxifen. They included 474 older women of 70 years of age and beyond and compared tamoxifen alone with a loading dose of 160 mg on day 1, followed for 5 years by 20 mg once a day or surgery followed by tamoxifen 20 mg daily for 5 years. They found no differences in overall survival after 6 years. However, progression-free survival was significantly better in the surgery with adjuvant tamoxifen group. Long-term follow-up yielded similar results.

The trial of Fennessy et al. [20] included 455 women aged 70 years or older. Their primary outcome was time to treatment failure (TTF). TTF was significantly shorter in the tamoxifen alone group. In addition, they showed that both overall survival and mortality from breast cancer were significantly increased in this group.

The study by Wilsher et al. that was previously mentioned randomised 147 women with ER-positive tumours to tamoxifen 20 mg once a day or mastectomy followed by tamoxifen 20 mg daily. Again, no differences were found in survival outcome between the two groups, and after 6 months, there was a complete response of 30% in the tamoxifen group. They concluded after both research portions that an approximately 60% failure rate makes primary endocrine therapy an inappropriate general treatment for elderly women who are fit for surgery. Controversially, they suggest an evaluation at 6 months and if women are in complete response or partial response with tamoxifen alone, they should continue just this treatment and if there is progression proceed to surgery.

More recently, Johnston et al. [21] reported on 20 years of follow-up of a randomised controlled trial of primary tamoxifen compared to mastectomy and adjuvant tamoxifen. They randomised 153 women of 70 years and older with breast cancer stadia T_1/2_, N_0/1_, and M_0_ and where fit to undergo surgery. They found no statistically significant difference in breast cancer-specific or overall survival. Also, they compared the rates of locoregional recurrence and metastases and found no differences.

Summarising, primary endocrine therapy compared to surgery with adjuvant endocrine therapy was shown to be an inferior treatment alternative, mostly because of the significantly improved progression-free survival and local control with dual therapy (HR 0.65, 95% CI 0.53–0.81, *P* = 0.0001). Concerning overall survival, there was no significant difference; however, the *P* value was 0.06 (HR 0.86, 95% CI 0.73–1.00) in the meta-analysis of Hind et al. [12]. Hence, combined therapy might be a better treatment in terms of overall survival outcomes.

## Observational Studies

Many observational studies have studied the effect of surgical treatment compared with primary endocrine treatment [22–24]. However, studying treatment effects in observational data is extremely challenging due to selection bias. For example, Morgan et al. [25•] analysed the data of 17.129 women of 70 years and older from 2002 to 2010 in the UK. Patients who received surgical treatment were likely to be younger, had lower stages of breast cancer and had fewer or even no comorbidities [25•].

Even after adjustment for confounders, it is likely that women who received primary endocrine treatment were more “frail” than women who received surgical treatment [26•].

More recently, Cortadellas et al. [27•] conducted a cohort study of 369 women of 80 years and older between primary tamoxifen and surgery alone. They showed that only women with early stage breast cancer (stages I, IIA, IIB) had better survival outcomes with surgery alone. Women with stage IIIA, IIIB, and IIIC breast cancer showed no difference in overall survival between tamoxifen and surgery. In both groups, primary endocrine therapy alone had an inferior progression-free survival compared to surgery. Cortadellas et al. [28] also compared 259 women again 80 years and older with primary endocrine therapy and surgery followed by endocrine therapy and reported the same overall survival in both groups while the surgery group had better breast cancer-specific survival.

Therefore, we only present studies here that have used methods that did not directly compare both treatment strategies, and are therefore not prone to bias.

Several studies have assessed changes in treatment strategies in relation to overall survival over the years. For example, Kantor et al. [29•] identified 95,357 women aged 80 years in the USA or older with invasive ER-positive breast cancer between 2004 and 2012. Ninety percent of these women were treated by surgery (alone or with adjuvant therapy) and only 10% had a non-operative treatment (50% primary endocrine therapy, 22% primary endocrine therapy eventually followed by surgery and 27% had no treatment). Interestingly, the study showed a significant increase in the rate of women who received primary endocrine therapy between 2004 and 2012 (from 7% in 2004 to 14% in 2012). This study did not assess the effect on survival outcomes.

Similarly, two Dutch studies of Hamaker et al. and De Glas et al. [30••, 31••] studied changes in surgical treatment strategies in a large cohort of 26,292 women of 75 years or older. They showed that the proportion of women who received surgical treatment significantly decreased between 1995 and 2011 (in 1995, 91% of women underwent surgery, versus 70% in 2011). Consequently, in 1995, 7% of older women received primary endocrine therapy versus 27.3% in 2011. Women who were not surgically treated were in general older and had advanced tumour stage and ER-positive tumours. Among the 86.8% of these women who did not receive breast surgery, endocrine therapy was prescribed while almost 2% did not receive any treatment at all. Even with these significant and strong changes in treatment strategy, no changes in overall survival nor relative survival were observed over time between 1995 and 2011.

Important reasons for omitting surgery have shown to be firstly patient choice, followed by comorbidity and age [23, 30••]. Wink et al. [22] identified the same reasons in a multicentre cohort of 184 women aged 75 years and older. Of these women, 92% were treated with primary endocrine therapy, while the remaining 8% did not receive any treatment at all. In this cohort, breast cancer was the primary cause of death in 14%, while 60% of patients died of other diseases and for 26%, the cause of death could not be retrieved. In comparison, Hamaker et al. found breast cancer to be the primary cause of death in only 34% of patients, while cardiac disease was the most frequent non-malignant cause of death, counting for 20% of the remaining patients. There was no difference in survival between women dying of their breast cancer versus dying from all other diseases [30••].

## Adverse Events, Functional Status and Quality of Life

A recent Dutch study showed that the risk of postoperative complications increases with age and comorbidity [32••]. Women aged 65–69 years had a lower rate of complications (15.3%) than women of 85+ years old (25.1%). The most frequent complications were haematomas and seromas. In addition, polypharmacy was a strong predictor for the risk of postoperative complications. The incidence of severe or life-threatening complications was 0% and was low. Similarly, a German study included 184 patients with breast cancer aged 80 years or older and showed that although the incidence of postoperative complications was 34.5%, these were rarely severe complications [33]. However, to our knowledge, no previous studies have examined the long-term physical functioning or quality of life after breast cancer surgery in older women. It can be hypothesised that especially lymphoedema or seroma can result in serious physical impairments [34].

At older age, endocrine therapy also comes with adverse events. Van de Water et al. [35] showed that 7.5% of women 65–74 years of age stop endocrine therapy in the first year, while in the age group of > 75 years, this percentage was even 13.2%. Adverse events and toxicity were the main reasons for discontinuation in 83.1 and 89.6% of patients, respectively. Among the adverse events were hot flashes, headache, venous thrombosis, depression, nausea, hair loss, vision problems, cystitis, vaginal bleeding and indigestion problems [12••, 36].

## Discussion

In this overview, we have shown that primary endocrine therapy compared with either surgery alone or surgery with adjuvant endocrine therapy results in worse local disease control and time to progression. When comparing surgery alone with primary endocrine therapy, no strong differences in terms of overall survival were observed in any of the studies, but surgery combined with adjuvant endocrine therapy might result in an improved overall survival (although not statistically significant). These findings were supported by observational studies. As shown, there were no (randomised controlled) trials performed in the most recent decade. Both surgical resection and endocrine therapy come with complications and side effects that occur more frequently with increasing age, but these are generally relatively mild. No data are available comparing the long-term quality of life or physical functioning between the two treatment approaches.

In 2012, the International Society of Geriatric Oncology (SIOG) and the EUSOMA published an update of the guideline for treatment of older women with breast cancer [7••]. They recommended that women of 70 years or older with early stage breast cancer should be treated the same as younger women with the standard of care being surgery. Regarding primary endocrine therapy, the 2012 guidelines recommend primary endocrine therapy as an accepted treatment for elderly women with specific characteristics, for example ER-positive tumours, short estimated life expectancy and being unfit for surgery [7••]. This was changed from the previous edition (2007) that stated that solely primary endocrine therapy was inferior to surgery and could not be not recommended at all.

An important limitation of the randomised trials that were described in this review is that all studies were performed with tamoxifen, although aromatase inhibitors or sequential treatment with tamoxifen followed by aromatase inhibitors can be considered the present standard in postmenopausal women as endocrine therapy. Recently, the British ESTEeM trial [37] was set out to compare an aromatase inhibitor with surgery (with or without adjuvant aromatase inhibitor), but poor patient inclusion resulted in premature closure of the study. In addition, no trials with sequential therapy were performed with solely elderly women. This limits the applicability of these trials in daily clinical practice, as there are nowadays several lines of endocrine therapy that can be used sequentially in case of treatment failure.

A second limitation of the trials that were mentioned was possible selective inclusion of patients. Van de Water et al. [10••] have previously shown that older women in breast cancer trials are not comparable with the general population in the consulting room. They showed that the study population had less comorbidities, a higher socio-economic status and lower stages of breast cancer. Therefore, an alternative way to assess treatment effects in older patients is observational research, provided that adequate research methods are used [26•].

With this review and with earlier recommendations, it could be argued that primary endocrine therapy might be a good and appropriate alternative for elderly women with breast cancer with multiple comorbidities and an increased risk of dying from another cause than the breast cancer. As shown in these studies, the majority of older women with breast cancer died from other causes than breast cancer [22, 23, 30••]. On the other hand, it has been shown that although the risk of postoperative complications increases with age, complications were relatively mild and did not result in an increased mortality risk [32••]. However, long-term effects of the omission of surgery remain unknown, especially in terms of functional status and quality of life. The observed poor local control might result in impaired functional status and quality of life. In addition, the effects of adverse events of endocrine therapy in older women on the long term are uncertain. It has been shown that a large proportion of older women have problems to endure the endocrine therapy and quit prematurely [35].

An important aspect in this decision process is the opinion of the patient. Burton et al. studied the information needs of older women with breast cancer [38•]. All patients wanted information on the impact of the treatment on independence, practicalities of treatment and healthcare professional’s recommendations. When patients were asked what treatment they preferred, there were two frequent answers: optimal disease control by surgery or minimal disruption of life by primary endocrine therapy [38•]. Another study investigated surgeon’s opinions and concluded that 93% of surgeons believed that primary endocrine therapy should be limited to elderly women who are not fit for surgery. However, 7% of the surgeons would recommend it for fit elderly women with operable breast cancer, because of risks of surgery and the impact on quality of life [39].

The key to making the decision between primary endocrine treatment and surgery is to differentiate between “fit” and “unfit” patients. The SIOG recommends performing a geriatric assessment (GA) in all older patients with cancer [8]. Girre et al. and Chiabi et al. [40, 41] showed that in half of the cases, changes in final treatment decisions were made after a geriatric assessment. In addition, Kenis et al. [42] showed that in 51% of women, the assessment detected unknown geriatric problems which influenced consequently the treatment decisions. A geriatric assessment provides information that is more relevant than age and performance status in determining frailty and survival expectation [43]. However, no studies have investigated how the geriatric assessment can determine which women should (or should not) receive surgical treatment. In addition, more studies are needed to assess long-term quality of life and functional status after surgery or primary endocrine therapy.

## Clinical Implications

**Fig. 1:**
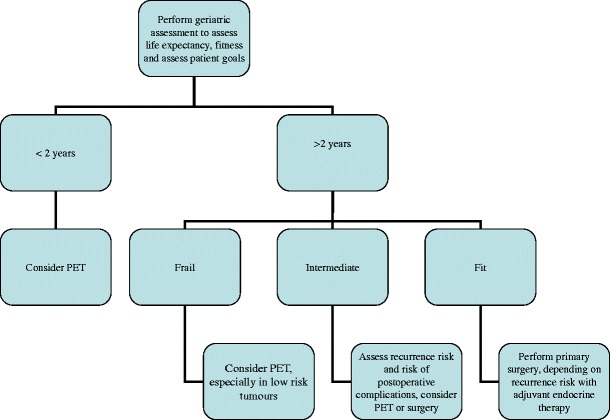
Flowchart of primary endocrine treatment

In daily clinical practice, we suggest performing a geriatric assessment in all older women in order to estimate remaining life expectancy. Especially if remaining life expectancy is limited, primary endocrine therapy might be the preferred treatment. In case of disease progression, several subsequent lines of endocrine therapy are available. If these fail or are contraindicated, surgical treatment can be performed at a later stage, without influencing survival outcomes. Still, the frequency of follow-up visits may be a factor in the treatment choice of patients. After surgery, patients have only a few follow-up visits, while endocrine therapy requires more frequent visits to monitor side effects and progression of disease. This may be a limiting factor in frail older patients and might be a reason to choose primary surgical treatment. In our opinion, primary endocrine treatment is a viable option especially for older women with multiple comorbidities and limited life expectancy, as seen in Fig. 1. Since an increasing proportion of patients prefer this treatment strategy, patients should be counselled on both options to enforce shared decision-making.

## Conclusion

Primary endocrine therapy results in reduced local control compared with surgical treatment, but no differences in overall survival are observed in randomised clinical trials nor observational studies. Long-term effects on quality of life and functional status remain unknown for both treatment strategies. The geriatric assessment may be used to tailor treatment and predict which women may be unfit for surgery or have a reduced remaining life expectancy and hence may be good candidates for primary endocrine therapy. Future studies should address selection of patients for either treatment strategy as well as quality of life and functional outcomes.

## Abbreviations

ER: Oestrogen receptor
EUSOMA: European Society of Breast Cancer Specialists
GA: Geriatric assessment
HR: Hazard ratio
PET: Primary endocrine therapy
SIOG: International Society for Geriatric Oncology
TTF: Time to treatment failure
